# Genetic alterations and aberrant hormonal pathways in thymic epithelial tumors

**DOI:** 10.1186/s12885-025-15455-4

**Published:** 2025-12-12

**Authors:** Yuqi Zhang, Wenxin Lian, Wenyan Zuo, Fufeng Wang, Yaru Zhang, Jing He, Yan Wang, Wen Gao

**Affiliations:** 1 Department of Oncology, The First Affiliated Hospital of Nanjing Medical University, Nanjing, Jiangsu China; 2 Nanjing Geneseeq Technology Inc Geneseeq Research Institute, Nanjing, Jiangsu China; 3 Endoscopy Center, The First Affiliated Hospital of Nanjing Medical University, Nanjing, Jiangsu China; 4 The Friendship Hospital of Ili Kazakh Autonomous Prefecture, Ili & Jiangsu Joint Institute of Health, Yining, Xinjiang China

**Keywords:** Thymic epithelial tumor, Exome sequencing, Hormone, Paraneoplastic syndromes, Gene mutation

## Abstract

**Background:**

Patients with thymic epithelial tumors (TETs) frequently show coexistence of various paraneoplastic syndromes, which severely affect their survival. Moreover, there is a lack of effective clinical treatment strategies for patients with unresectable metastatic and recurrent TETs.

**Methods:**

To explore the genetic alterations that play a key role in the pathogenesis of TETs, we analyzed the whole-exome sequencing data from 24 patients diagnosed to have TETs at the First Affiliated Hospital of Nanjing Medical University.

**Results:**

Mutated genes in TETs were enriched in several hormone-associated pathways such as insulin secretion; Cushing syndrome; parathyroid hormone; and thyroid hormone, as well as multiple classical tumor-associated pathways, including cAMP, Notch, PI3K-Akt, and WNT signaling pathway. Patients with paraneoplastic syndromes (PNS) exhibited more pronounced alterations in hormone-related pathways. *RHPN2* is the most frequently mutated gene in TETs. TETs with *RHPN2* mutation showed greater upregulation of the hormone-related signaling pathways such as thyroid hormone and parathyroid hormone as well as a trend toward shorter survival of patients.

**Conclusion:**

We analyzed the possible role of hormones in TETs on several levels, explored potential links between hormones and other genetic mutations, and found that *RHPN2* may be a potentially valuable gene.

**Supplementary Information:**

The online version contains supplementary material available at 10.1186/s12885-025-15455-4.

## Introduction

Thymic epithelial tumors (TETs), including thymomas (THYM) and thymic carcinomas (TC), are a rare type of tumors that originate from thymic epithelial cells; however, these tumors are also the most frequent malignancies that develop in the anterior mediastinum in humans, with an annual incidence rate of 1.3 to 3.2 cases per million individuals [1–3]. Patients with TETs frequently have other coexisting illnesses, such as various autoimmune diseases and paraneoplastic syndromes (PNS), which severely affect their quality of life and survival and often lead to poor prognosis [2, 4]. PNS are commonly associated with altered hormonal functions [5, 6]; however, to date, few relevant studies have reported the role of both altered hormonal functions and PNS in patients with TETs.

Surgery is the primary treatment for TETs [4]. For patients with metastatic and recurrent nonresectable tumors, treatment with chemotherapeutic agents, such as cisplatin combined with anthracyclines or paclitaxel, is the first choice; however, this approach shows limited efficacy, with severe adverse effects. Targeted therapies also exhibit poor efficacy; moreover, the lack of sufficient information regarding genomic alterations in TETs hinders the development of targeted therapies [2, 7–9]. Although immune checkpoint inhibitors (ICIs), such as programmed death 1 (PD-1) and its ligand (programmed death ligand-1 (PD-L1)) inhibitors, have revolutionized tumor therapy, a clinical study showed that only 35.7% of patients with high PD-L1 expression achieved partial remission, and the high frequency of severe immune-related adverse events associated with these inhibitors limited their application in treating TETs [2, 10, 11].

Clinical dissatisfaction with the current state of diagnosis and treatment of TETs is mainly due to the lack of understanding of their mutations at the molecular level. The rarity of TET incidence limits the sample size required for research; additionally, the lack of established in vitro and in vivo models hinders the use of validated preclinical models and high-quality evidence for reference. Although some previous studies have analyzed TETs at the molecular level, they generally focused on THYM alone and reported inconsistent findings. Given the limitations of the diagnostic and therapeutic strategies for TETs, it is critical to conduct further research on mutations in TETs at the molecular level.

In the present study, to provide an appropriate reference for the diagnosis and treatment of TETs, we examined the genetic panorama of TETs at the genomic level by conducting whole-exome sequencing (WES) to determine genetic alterations that play a key role in TET development and analyzed the hormonal changes occurring in TETs.

## Methods

### Patient details and sample collection

This study included 24 Chinese patients diagnosed with TETs (18 patients with TC and 6 patients with THYM) at the First Affiliated Hospital of Nanjing Medical University from 2019 to 2024. Tumor tissues and whole blood control samples from the patients were analyzed by WES at Nanjing Geneseeq Technology Inc. The clinical data of the patients were obtained from electronic hospital records. TC or THYM diagnosis was confirmed by two experienced pathologists according to the 2021 World Health Organization’s classification. This study was conducted in accordance with the Declaration of Helsinki and was approved by the Ethics Committee of the First Affiliated Hospital of Nanjing Medical University (Approval No. 2025-SR-496). Written informed consent was obtained from each patient before sample collection.

### Whole-exome library preparation and sequencing

Genomic DNAs were extracted from formalin-fixed paraffin-embedded (FFPE) sections or biopsy samples and whole blood control samples by using the QIAamp DNA FFPE Tissue Kit (Qiagen) and the DNeasy Blood and Tissue Kit (Qiagen), respectively, and quantified by Qubit 3.0 using the dsDNA HS Assay Kit (Thermo Fisher Scientific). A whole-genome library was constructed using the KAPA Hyper Prep Kit (KAPA Biosystems). Whole-exome capture was performed using the xGen™ Exome Hybridization Panel (Integrated DNA Technologies, Inc.) in accordance with the manufacturer’s protocol. The enriched libraries were then sequenced on an Illumina HiSeq4000 sequencing platform (Illumina) using the PE150 sequencing strategy. The average coverage depth was 354× and 144× for tumor cells and white blood cell controls, respectively.

### Variant filtering and mutation calling

Trimmomatic was used for the quality control of FASTQ files [12]. Leading/trailing low-quality bases (quality reading below 20) or N bases were removed. Burrows-Wheeler aligner was then used to align clean paired-end reads to the reference human genome (hs37d5) [13]. PCR deduplication was performed using the Picard tool (Broad Institute), and local realignment around insertion/deletions (indels) and base quality score recalibration were performed using the Genome Analysis Toolkit (GATK v 3.4.0) [14]. Samples with a mean depth of < 30× were also removed. Somatic single nucleotide variant and indel calling was performed using VarDict v 1.5.4, and the results were filtered using the following criteria: (i) filtered if variant-supporting reads < 4 or variant allele frequency (VAF) supporting the variant is < 2%; (ii) filtered if present in >1% population frequency in the 1000 genomes or ExAC database [15, 16]; and (iii) filtered through an internally collected list of recurrent sequencing errors (≥ 3 variant reads and ≤ 20% VAF in at least 30 of ~ 2000 normal samples) on the same sequencing platform. The final list of mutations was annotated using vcf2maf (call VEP for annotation).

Tumor mutation burden (TMB) was defined as the total number of nonsynonymous mutations divided by the length of the genomic target region [17]. Microsatellite instability (MSI) was detected using the MSI-sensor2 tool v 0.1 (https://github.com/niu-lab/msisensor2) [18]. Tumor samples with a count of ≥ 5 synonymous/nonsynonymous mutations were included in the mutation signature analysis using maftools and sigminer packages in R software (v 4.1.3) [19].

### Identification of driver mutation

MutSigCV was used to identify significantly mutated genes [20]. MutSigCV considers the overall mutation condition of the genome, mutation frequency of genes near the mutation site, location of the site in a region where the chromosome can easily open, and other relevant parameters to find genes with a mutation rate higher than the calculated background mutation rate. Multiple test correction (Benjamin–Hochberg false discovery rate [FDR]) was performed, and genes with a *q* value of < 0.1 were reported. We defined potential driver mutations if one of the following conditions was met: (i) mutations are documented in the COSMIC database and (ii) mutation frequency is >3 in our dataset.

### Copy number analysis

Significantly amplified or deleted focal copy number variation (CNV) regions and potential cancer driver genes were identified using Genome Identification of Significant Targets in Cancer (GISTIC) 2.0 [21]. Chromosome arms were labeled as “altered” in each cohort if the GISTIC q value was < 0.15.

### Pathway enrichment analysis

The 50 Hallmark gene set was downloaded from the Molecular Signatures Database (MsigDB) [22]. The clusterProfiler package was used to analyze and compare variations in oncogenic pathway activities derived from both the Kyoto Encyclopedia of Genes and Genomes (KEGG) database and the MSigDB between different groups. A *P-*value of < 0.05 was considered statistically significant.

We downloaded immune-related genes from the Immunology Database and Analysis Portal (ImmPort) (https://www.immport.org/home), which contains 2498 immune-related genes from 17 immune gene categories. Fisher’s test was used to compare differences in immune-related pathways between different groups.

### Statistical analysis

All statistical analyses were performed in R (version 4.1.3). Fisher’s exact test was used to determine associations between categorical variables. The Wilcoxon rank-sum test was used to compare medians of independent groups. Kaplan-Meier survival curves were generated to analyze the overall survival of different patient groups. Statistical differences were assessed using the log-rank test. Statistical significance was set at *P* ≤ 0.05.

## Results

### Clinical characteristics of study patients (Table 1)

Our study comprised 24 patients with TETs; of these, 18 and 6 patients had TC and THYM, respectively. According to the Masaoka staging system, all patients were diagnosed with advanced-stage disease: one (4.2%) with stage IIIA, one (4.2%) with stage IIIB, seven (29.2%) with stage IVA, and fifteen (62.5%) with stage IVB. The mean age of the cohort was 59 years (26–73 years). The male: female ratio was 58.3%:41.7%. As of June 2024, the median progression-free survival (PFS) of all patients was 6.5 months. The mean age of patients in the TC cohort was greater than that in the THYM cohort (*P* = 0.198). The male: female ratio in the TC cohort (11:7) was similar to that in the THYM cohort (3:3) (*P* = 0.665). Paraneoplastic syndromes were observed in 8 patients (33.3%), including leukocytosis in 3 (12.5%), elevated cortisol in 1 (4.2%), thyroid dysfunction in 1 (4.2%), autoimmune disease in 1 (4.2%), and myasthenia gravis in 3 (12.5%). One patient presented with both leukocytosis and elevated cortisol.

**Table 1:** Characteristics of patients

Characteristics	Total = 24
Age at diagnosis	
Median age(range).years	59 (26–73)
Gender	
Female	10 (41.7%)
Male	14 (58.3%)
Histology	
Thymic carcinoma	18 (75.0%)
Thymoma	6 (25.0%)
Masaoka stage	
IIIA	1 (4.2%)
IIIB	1 (4.2%)
IVA	7 (29.2%)
IVB	15 (62.5%)
Median progression-free survival.months	6.5
Paraneoplastic Syndromes	8 (33.3%)
Leukocytosis	3 (12.5%)
Elevated cortisol levels	1 (4.2%)
Thyroid dysfunction	1 (4.2%)
Autoimmune disease	1 (4.2%)
Myasthenia gravis	3 (12.5%)

### Genomic characteristics of study patients

**Fig. 1:**
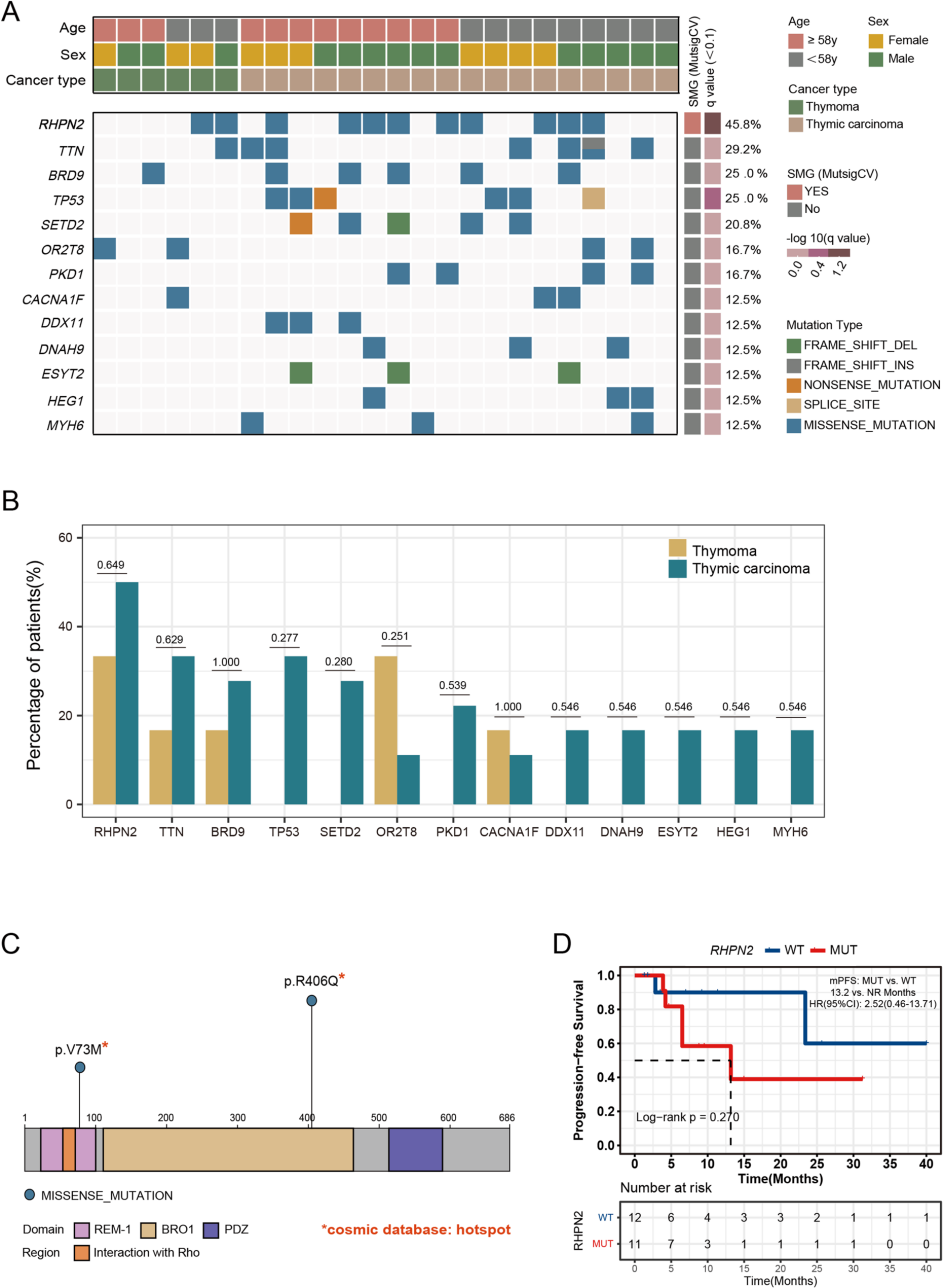
*RHPN2* as a potential key gene in thymic epithelial tumors (TETs). **A** The top 14 mutations in the thymic carcinoma (TC) and thymoma (THYM) cohorts. **B **Comparison of mutated genes in TC and THYM. **C** Annotation based on the COSMIC database revealed that all *RHPN2* gene mutations were located in the hotspot region. **D** Correlation between the *RHPN2* gene and progression-free survival (PFS) of patients with TETs. Abbreviations: WT, wild type; MUT, mutant type; mPFS, median progression-free survival

We detected 1320 nonsynonymous mutations in tumor tissue samples from 24 patients with TETs. Analysis of the overall data showed that *RHPN2* was the most frequently mutated gene in TETs, with a mutation rate of 45.8% (*n* = 11). Other high-frequency mutations included *TTN* (29.2%), *BRD9* (25.0%), *TP53* (25.0%), and *SETD2* (20.8%) (Fig. 1A).

We then individually analyzed the TC and THYM cohorts. In the TC cohort, the top three high-frequency mutations were *RHPN2* (50.0%, *n* = 9), *TP53* (33.3%, *n* = 6), and *TTN* (33.3%, *n* = 6); in the THYM cohort, the top three high-frequency mutations were *RHPN2* (33.3%, *n* = 2), *KNDC1* (33.3%, *n* = 2), and *OR2T8* (33.3%, *n* = 2). The TC and THYM cohorts showed no significant differences in high-frequency mutations (Fig. 1B).

**Fig. 2:**
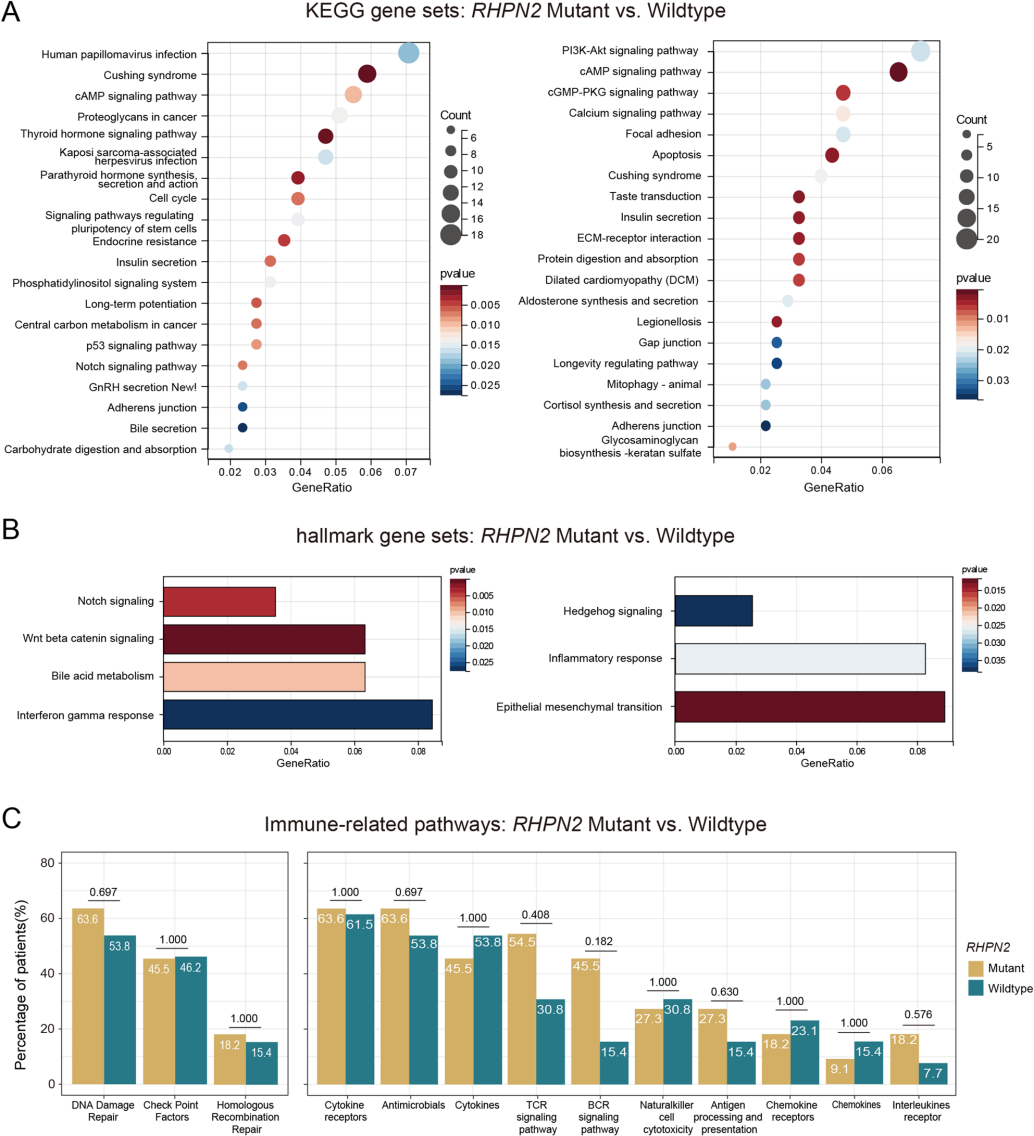
Differences in gene enrichment between *RHPN2* mutant and wild type. **A** Analysis based on the gene set derived from the Kyoto Encyclopedia of Genes and Genomes (KEGG) database, which shows the top 20 pathways with *p* < 0.05. **B** Analysis based on the gene set derived from the Hallmark database. **C** Analysis of the immune-related pathways based on the gene set derived from the Immunology Database and Analysis Portal (ImmPort)

Based on the somatic mutation profiles of 24 patients with TETs, we identified *RHPN2* as the most significant mutated gene by using the MutSigCV algorithm *(P* < 0.05; q < 0.1). Annotation based on the COSMIC database revealed that all *RHPN2* gene mutations were located with known hotspot region, including p.V73M in 3 (12.5%) patients and R406Q in 8 (33.3%) patients (Fig. 1C). We also analyzed the correlation between the *RHPN2* mutated gene and PFS of patients with TETs. The results showed that TET patients with *RHPN2* mutation had a shorter PFS (13.2 months vs. NR; HR (95% CI): 2.52 (0.46–13.71); *P* = 0.270); however, a significant difference was not observed because of the limited sample size and short follow-up duration (Fig. 1D). Moreover, TETs with *RHPN2* mutation showed a greater mutations in oncogenic pathways such as the Notch and WNT pathways as well as hormone-associated pathways such as thyroid hormone and parathyroid hormone (*P* < 0.05) (Fig. 2A-C). Following FDR correction, alterations in the thyroid hormone signaling pathway and WNT pathway in *RHPN2*-mutant TETs still exhibited significant differences (*P* < 0.05) (Figure S1 A-B), and the complete results are available in Supplementary Table S1 (Table S1).

**Fig. 3:**
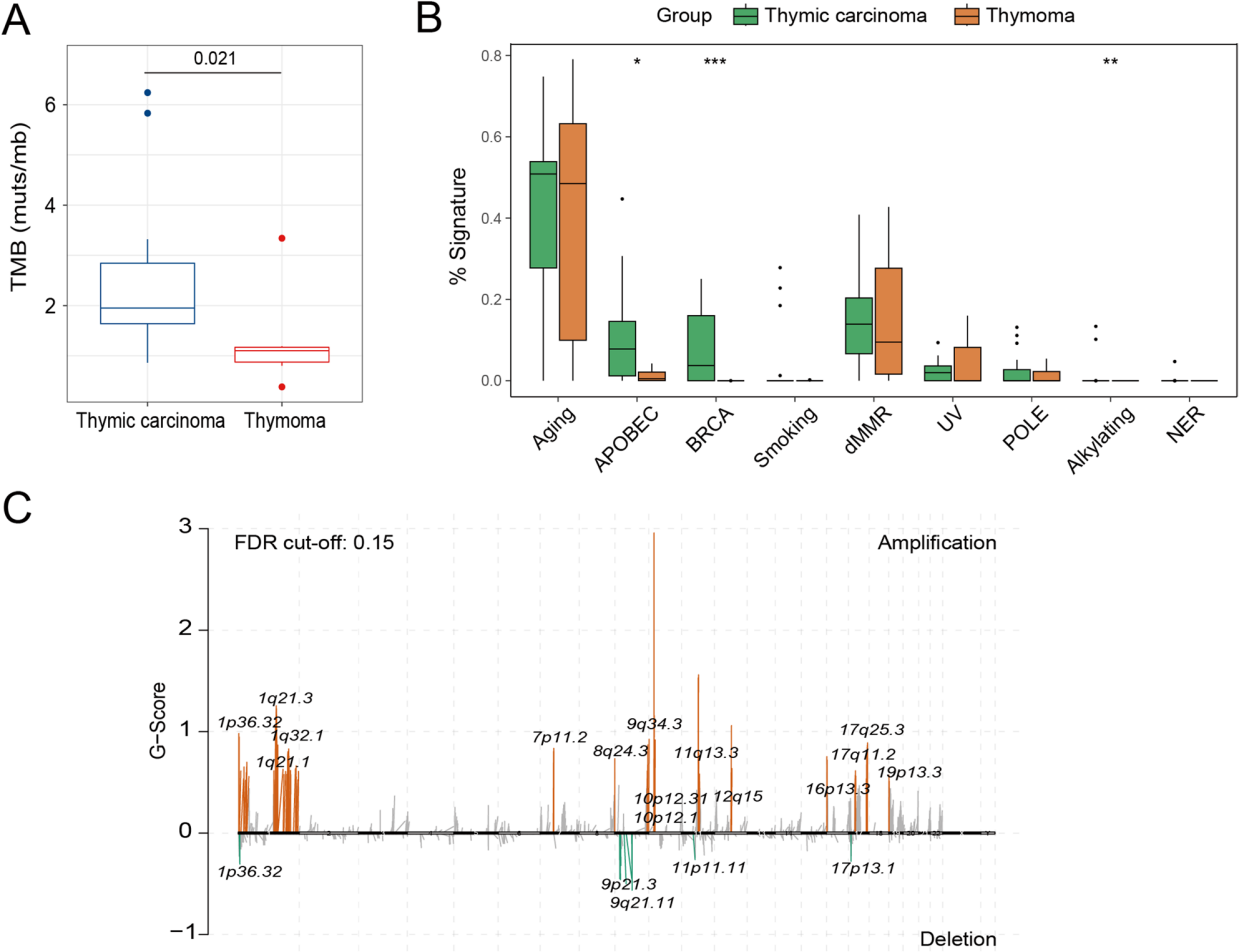
Characteristics of thymic epithelial tumors (TETs) in terms of tumor mutation burden (TMB), mutation signatures, and copy number variation (CNV). **A** The median TMB value of thymic carcinoma (TC) and thymoma (THYM). **B** The mutation signatures of TC and THYM. **C** Recurrent CNV events in TETs

We further analyzed the characteristics of TETs in terms of TMB and mutation signatures. The results revealed that the 18 TC samples had an average of 62.4 mutations (1123/18), the 6 THYM samples had an average of 32.8 mutations (197/6), and the median TMB value of TETs was 1.90 (0.38–6.24) and was significantly higher in TC patients than in THYM patients (1.95 vs. 1.1) (Fig. 3A). The mutation signatures of TETs were predominantly aging and DNA mismatch repair, and the signatures associated with APOBEC and BRCA were more enriched in the TC cohort (Fig. 3B).

Next, we delineated the recurrent CNV events by using the GISTIC 2.0 algorithm. At the focal CNVs, 21 peak regions were detected (q value < 0.15), with more amplifications than deletions (16 versus 5). The recurrent deletions included several known tumor suppressor genes (*CDKN2A*, *CDKN2B*, and *MTAP*). Other cancer genes detected in focal CNV peaks included *EGFR* (7p11.2 amplification), *MDM2* (12q15 amplification), *CCND1* (11q13.3 amplification), and *MLLT10* (10p12.31 amplification), which were also enriched in TETs (Fig. 3C).

Further analysis of the data revealed that 45.8% of patients with TETs carried potential clinical genetic mutations involving *PIK3ca*, *EGFR*, *CDK*, and *ERBB2*, which could be used as targets for drug therapy. The percentages of carriers in the TC and THYM groups were 50.0% and 33.3%, respectively (*P* = 0.649) (Table 2).

**Table 2:** Potential clinical genetic mutations carried by patients with TETs

PID	Cancer type	Hugo_Symbol	HGVSp_Short	level of OncoKB	Level-associated cancer types	Drugs
P09	TC	CDK12	Amplification	1/4	Prostate Cancer/ All Solid Tumors	PARP inhibitors/ICI
P12	TC	CDK12	Amplification	1/4	Prostate Cancer/ All Solid Tumors	PARP inhibitors/ICI
P10	TC	CDK12	Amplification	1/4	Prostate Cancer/ All Solid Tumors	PARP inhibitors/ICI
P12	TC	CDKN2A	p.V82Rfs*44	4	All Solid Tumors	Abemaciclib, Palbociclib, Ribociclib
P21	TC	CDKN2A	Deletion	4	All Solid Tumors	Abemaciclib, Palbociclib, Ribociclib
P25	TC	CDKN2A	Deletion	4	All Solid Tumors	Abemaciclib, Palbociclib, Ribociclib
P18	TC	CDKN2A	Deletion	4	All Solid Tumors	Abemaciclib, Palbociclib, Ribociclib
P25	TC	EGFR	Amplification	4	Glioma	Lapatinib
P09	TC	ERBB2	Amplification	1	Breast Cancer	Ado-Trastuzumab Emtansine
P12	TC	ERBB2	Amplification	1	Breast Cancer	Ado-Trastuzumab Emtansine
P10	TC	ERBB2	Amplification	1	Breast Cancer	Ado-Trastuzumab Emtansine
P02	TC	FGFR3	p.K650E	2/4	Bladder Cancer/ All Solid Tumors	Erdafitinib/AZD4547, Erdafitinib
P08	TC	KIT	p.Y823H	1	Gastrointestinal Stromal Tumor	Imatinib
P06	TC	MDM2	Amplification	3 A/4	Biliary Tract Cancer/All Solid Tumors	Ezabenlimab + Brigimadlin, Brigimadlin/Brigimadlin
P06	TC	PIK3CA	p.E542Q	1	Breast Cancer	Alpelisib + Fulvestrant
P02	TC	PIK3CA	Amplification	1/4	Breast Cancer/ All Solid Tumors	Alpelisib + Fulvestrant/RLY-2608
P04	THYM	AKT1	Amplification	2	Breast cacner	Capivasertib + Fulvestrant
P04	THYM	CDK12	Amplification	1/4	Prostate Cancer/ All Solid Tumors	PARP inhibitors/ICI
P04	THYM	ERBB2	Amplification	1	Breast Cancer	Ado-Trastuzumab Emtansine
P20	THYM	STK11	Deletion	4	Non-Small Cell Lung Cancer	Pembrolizumab + Bemcentinib

### Genomic pathway analysis

**Fig. 4:**
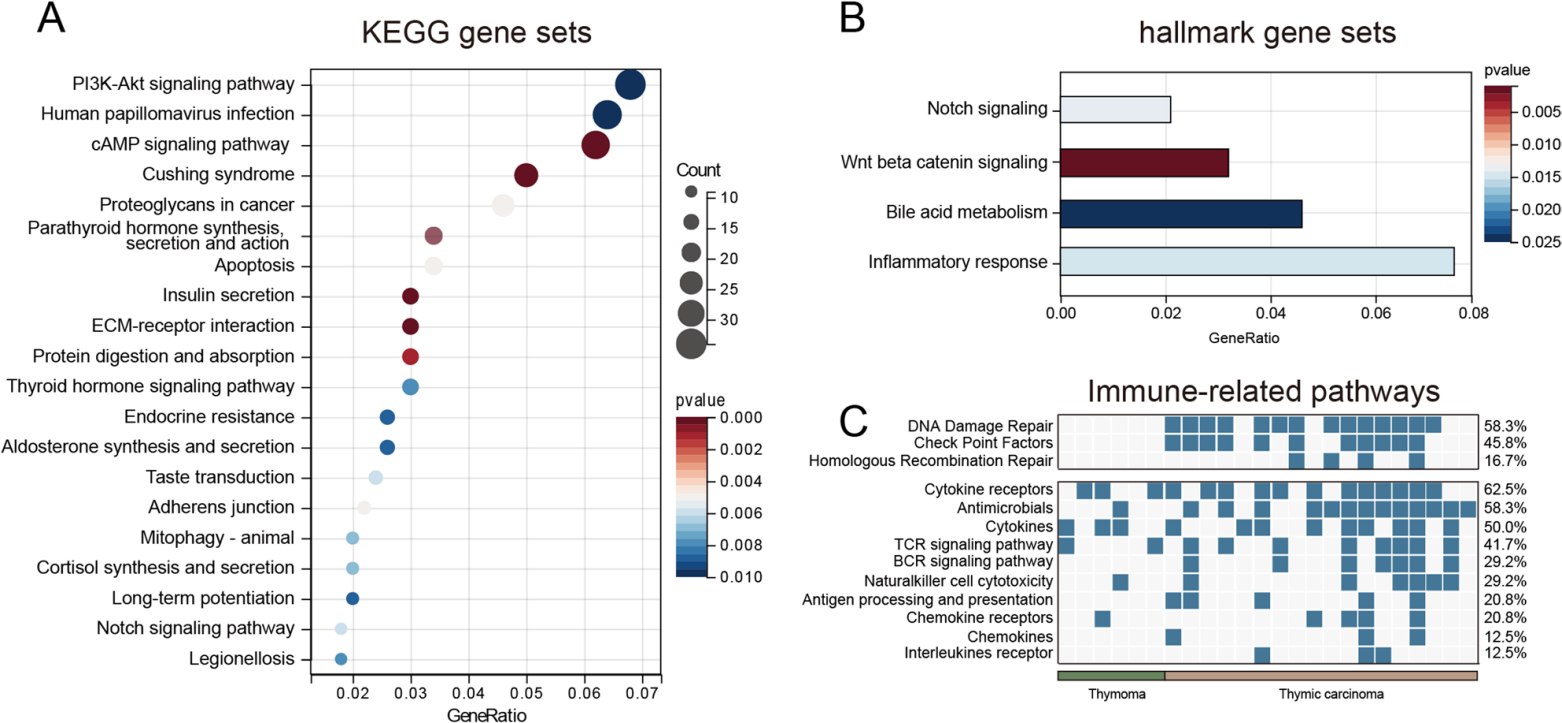
Gene enrichment of thymic epithelial tumors (TETs) in 24 patients. **A** Analysis based on the gene set derived from the Kyoto Encyclopedia of Genes and Genomes (KEGG) database, which shows the top 20 pathways with *p* < 0.05. **B** Analysis based on the gene set derived from the Hallmark database. **C** Analysis of the immune-related pathways based on the gene set derived from the Immunology Database and Analysis Portal (ImmPort)

**Fig. 5:**
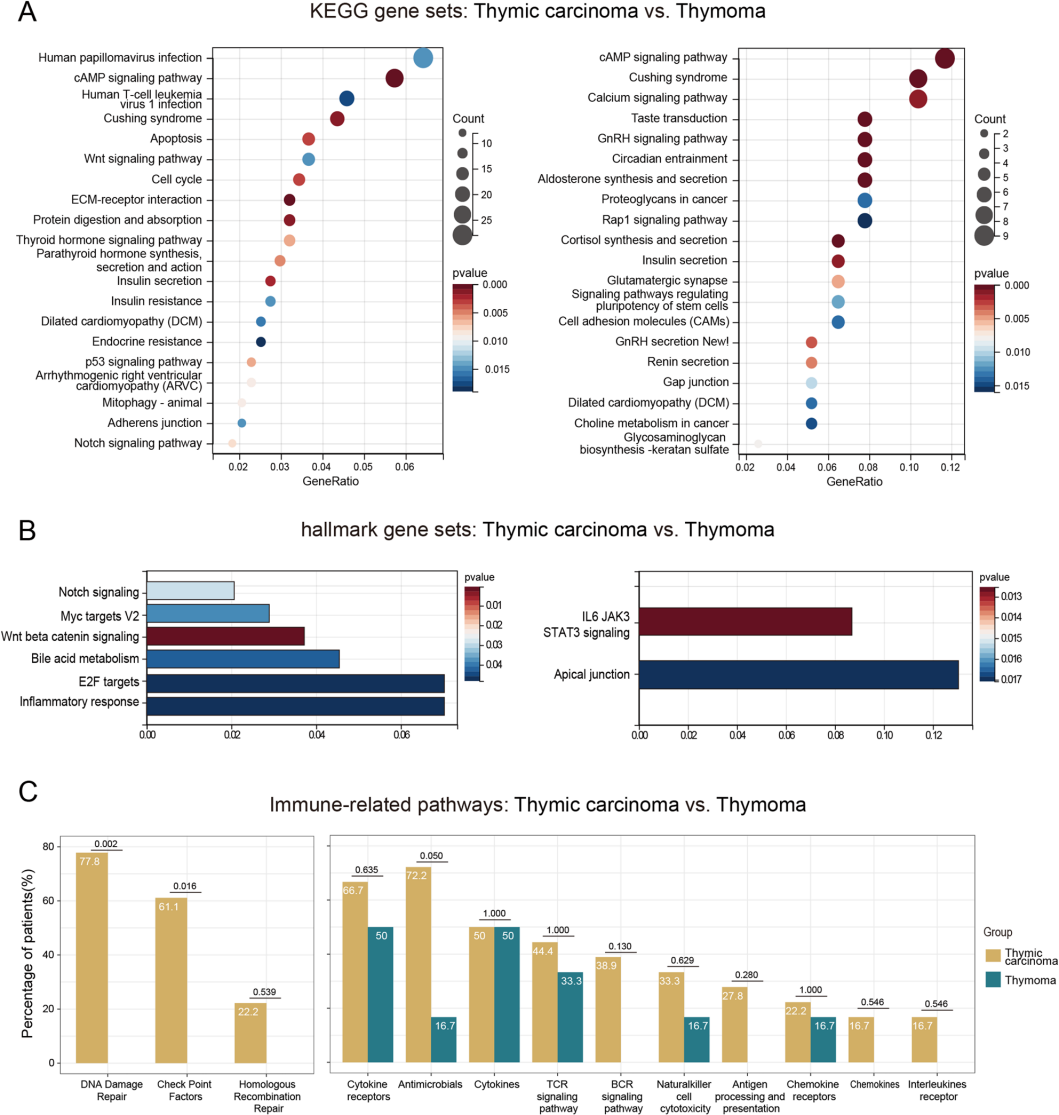
Differences in gene enrichment between thymic carcinoma (TC) and thymoma (THYM). **A** Analysis based on the gene set derived from the Kyoto Encyclopedia of Genes and Genomes (KEGG) database, which shows the top 20 pathways with *p* < 0.05. **B** Analysis based on the gene set derived from the Hallmark database. **C **Analysis of the immune-related pathways based on the gene set derived from the Immunology Database and Analysis Portal (ImmPort)

Based on the gene set derived from the KEGG database, we analyzed the gene enrichment for the genes associated with TETs in the 24 patients. The results showed that the genes of TETs were enriched in several classical tumor-related pathways such as cAMP signaling pathway, Notch signaling pathway, PI3K-Akt signaling pathway, and apoptosis (*P* < 0.05) (Fig. 4A). Following FDR correction, the cAMP pathway still exhibited significant alterations (*P* < 0.05). The mutant genes were also enriched in the Notch pathway and PI3K pathway (*P* < 0.2) (Figure S2 A). And the complete FDR correction results are available in Supplementary Table S1 (Table S1). This finding was also confirmed by the results of the gene set Hallmark analysis, which showed that the genes were enriched in the Notch signaling pathway and other signaling pathways such as the WNT/β-catenin signaling pathway and inflammatory response (*P* < 0.05) (Fig. 4B). Moreover, following FDR correction, the WNT pathway still exhibited alterations (*P* = 0.053) (Figure S2 B), and the complete FDR correction results are available in Supplementary Table S1 (Table S1). The KEGG pathway enrichment analysis revealed that the genes were enriched in multiple signaling pathways related to hormone synthesis and secretion, including Cushing syndrome; insulin secretion; parathyroid hormone synthesis, secretion, and action; and thyroid hormone (*P* < 0.05) (Fig. 4A). Additionally, after FDR correction, the aforementioned hormonal pathways still exhibited significant alterations (*P* < 0.05), except for thyroid hormones signaling pathway (*P* < 0.2) (Figure S2). The complete FDR correction results are available in Supplementary Table S1 (Table S1). We also found differences in gene pathways between the THYM and TC cohorts. Various signaling pathways, such as Notch, Wnt, thyroid hormone, parathyroid hormone, and endocrine resistance, were predominantly observed in the TC cohort as compared to that in the THYM cohort (Fig. 5A-B). Following FDR correction, the WNT pathway in the TC cohort showed significant alterations (*P* < 0.05); the results also indicated alterations in the Notch pathway, thyroid hormone pathway, and parathyroid hormone pathway (*P* < 0.2) (Figure S3 A-B). The complete FDR correction results are available in Supplementary Table S1 (Table S1).

**Fig. 6:**
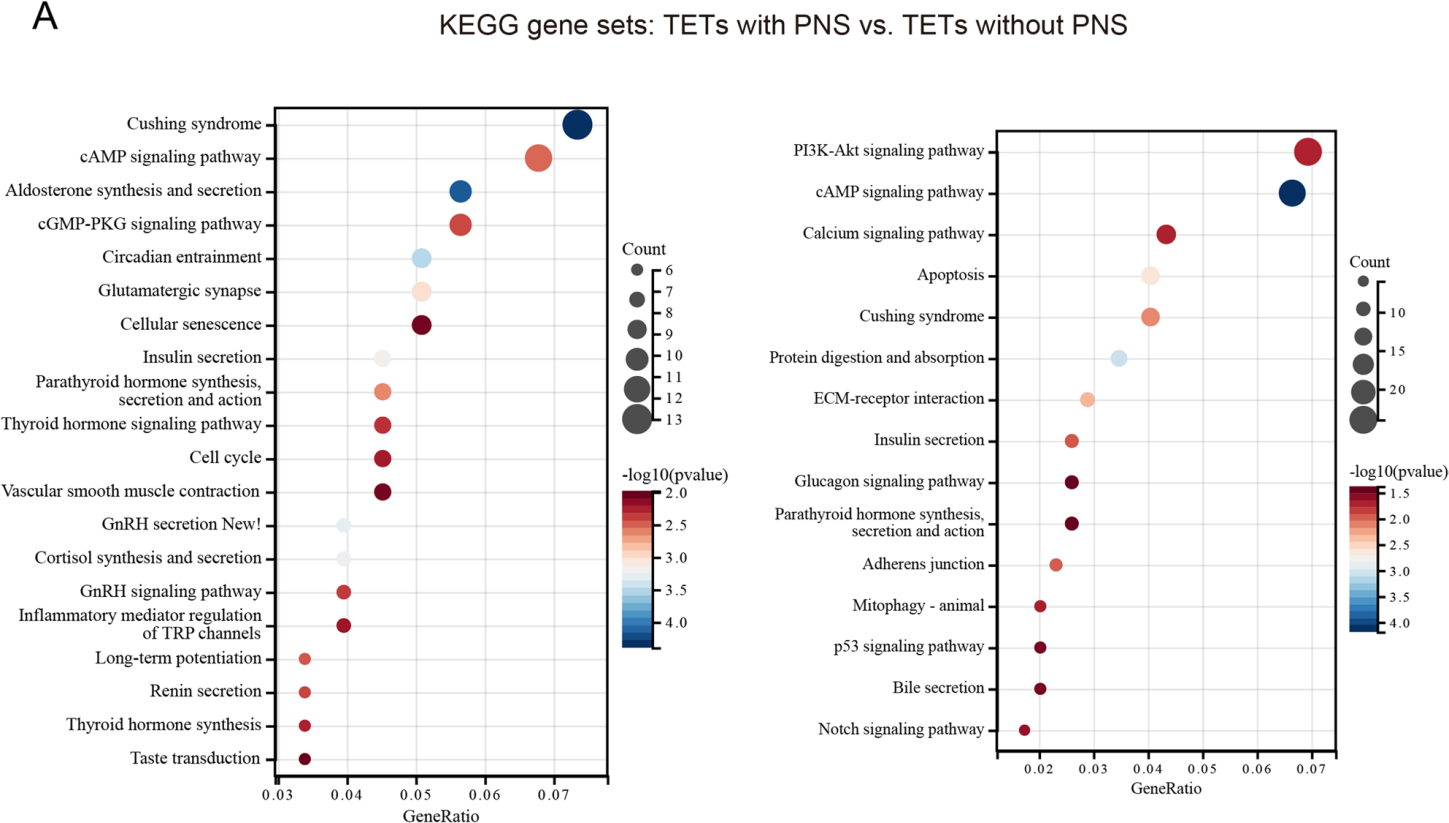
Comparative analysis of gene enrichment in TETs with and without paraneoplastic syndromes (PNS). **A** Analysis based on the gene set derived from the Kyoto Encyclopedia of Genes and Genomes (KEGG) database, which shows the top 20 pathways with *p* < 0.05

Furthermore, we stratified patients into two groups based on the presence or absence of PNS and performed KEGG pathway enrichment analysis. Results revealed that patients with PNS exhibited more pronounced alterations in hormone-related pathways, including Cushing syndrome, aldosterone synthesis and secretion, insulin secretion, parathyroid hormone synthesis, secretion and action, thyroid hormone signaling pathway, and thyroid hormone synthesis (*P* < 0.05). These pathways remained significantly altered even after FDR correction, with Cushing syndrome, aldosterone synthesis and secretion, and insulin secretion showing *P.adjust* < 0.05, and the remaining pathways displaying *P.adjust* < 0.08. In contrast, among patients without clinically apparent PNS, only Cushing syndrome, insulin secretion, and parathyroid hormone synthesis, secretion and action showed significant changes (*P* < 0.05), and none remained significant after FDR correction (*P.adjust* > 0.3) (Fig. 6). The complete FDR correction results are available in Supplementary Table S2 (Table S2).

Based on the data from the ImmPort database, we found that genetic mutations in TET patients occurred in multiple immune-related pathways, including DNA damage repair, cytokine receptors, antimicrobials, and cytokines (*P* < 0.05) (Fig. 4C). The incidence of mutations in the DNA damage repair (DDR) pathway (including checkpoint factor pathway) was significantly higher in the TC cohort (77.8%, 14/18) than in the THYM cohort (0.0%, 0/6) (*P* < 0.05) (Fig. 5C).

## Discussion

In the present study, we explored the gene mutational landscape in TETs, including THYM and TC, by conducting WES. We studied genetic alterations and aberrant hormonal pathways that may be associated with the development of TETs or PNS and determined the possible links between these aberrant hormonal pathways and other genetic alterations.

PNS are highly prevalent in TETs and are often mediated by hormonal dysregulation [5, 23]. Consistent with this observation, our pathway analysis revealed significant enrichment of mutations in genes involved in key hormone-related pathways, including insulin secretion, Cushing syndrome, parathyroid hormone, and thyroid hormone. These hormonal pathways have defined roles in tumor biology. For instance, the thyroid hormone-related pathway can promote tumor progression through the WNT and integrin avβ3 signaling pathway [24]. Regarding the pathways associated with parathyroid hormones, tumor cells can secrete various parathyroid hormone-related proteins to induce tumor progression; this feature is associated with tumor malignancy, poor prognosis, and shorter patient survival [25, 26]. In TETs, the activation of these pathways likely contributes to tumorigenesis. In addition to their oncogenic effects, these hormone-related pathways in TETs are also associated with various PNS, such as hypercalcemia, Cushing syndrome, and hypoglycemia [23]. These endocrine alterations may also influence the response and toxicity profiles of treatments such as immunotherapy, although this speculation requires further investigations for confirmation. We also found that hormone-related signaling pathways, such as thyroid hormone, parathyroid hormone, and endocrine resistance, were predominantly observed in the TC cohort as compared to that in the THYM cohort, which may be related to the relatively higher malignancy of TC. Moreover, we observed more pronounced alterations in hormone-related pathways—including Cushing syndrome, aldosterone, insulin secretion, parathyroid hormone, and thyroid hormone—in patients presenting with PNS. These findings further reinforce a robust association between the high incidence of PNS in TETs and the alterations in these hormone-related pathways. In patients with malignant tumors, PNS are usually associated with poor survival outcomes; thus, effective and prompt diagnosis and treatment of PNS can greatly improve clinical outcomes. We hope that the results of the present study could serve as a reference for the subsequent research, diagnosis, and treatment of TETs at the hormonal level.

Additionally, we noted that TET-associated genes were enriched in several classical tumor-related pathways such as cAMP, Notch, PI3K-Akt, and WNT signaling. These signaling pathways are considered to be associated with tumor proliferation, survival, migration, and immune evasion and exhibit tumor-promoting properties [27–32]. Especially, everolimus, an mTOR inhibitor targeting the PI3K-mTOR pathway, has been demonstrated to have therapeutic value in phase II clinical studies of thymic tumors [9]. Notably, these pathways do not act in isolation; according to previous literature, these pathways crosstalk with the hormonal pathways observed to be enriched in our cohort, including thyroid hormone, parathyroid hormone, and insulin secretion pathways [33–40]. cAMP is associated with several endocrine pathways, including the insulin secretion pathway, and is frequently dysregulated in various endocrine tumors. It can also promote immune evasion by reprogramming the tumor microenvironment and is associated with the progression of autoimmune diseases [27]. The PI3K pathway is an insulin-related pathway that promotes tumor development by altering glucose availability [41], while the Wnt pathway is inhibited by thyroid hormone receptors, resulting in tumor suppression [24]. The convergence of these pathways in TETs suggests a coordinated network that may simultaneously promote tumorigenesis and hormonal dysregulation, offering a unified framework to understand TET biology and its associated PNS. To date, few studies have addressed the link between these classical tumor-associated pathways and hormonal function. Thus, this finding further suggests the potential of hormonal intervention as a therapeutic strategy, which could assist in antitumor therapy and reduce the incidence of PNS.

Another key finding is the high mutation frequency of *RHPN2*. In addition to well-defined driver variants such as *TP53*, our study newly revealed that *RHPN2* is the most frequently mutated gene in TETs. According to the annotations based on the COSMIC database, we found that the *RHPN2* gene is involved in the G13 pathway, which plays an important role in the migration of fibroblasts and endothelial cells. Previous studies have shown that *RHPN2* can promote the malignant behavior of tumors through the RhoA and STAT3 pathways, leading to poor prognosis [42–44]. Compared to the wild-type gene, the mutant *RHPN2* gene showed a higher association with oncogenic pathways such as the Notch and WNT pathways as well as hormone-associated pathways such as thyroid hormone and parathyroid hormone. A previous study reported that STAT3 can activate the thyroid hormone signaling pathway, suggesting a potential mechanism through which RHPN2 influences hormone-related signaling pathways [42]; this finding warrants further validation in subsequent studies. Moreover, TET patients with the *RHPN2* mutant type had a shorter PFS. Although the difference in PFS between *RHPN2*-mutant and *RHPN2*-wild-type patients was not significant (*P* = 0.270)—likely due to the limited sample size and a short follow-up duration—we observed a trend toward shorter PFS in *RHPN2*-mutant patients. This observation suggests that *RHPN2* may have a prognostic value, which requires further validation in a larger cohort. This result suggests a potential role of *RHPN2* and the aberrant hormonal pathways in TETs as well as the possibility of a relationship between the two. A noteworthy finding is that TET patients carrying the *RHPN2* mutant gene may exhibit more number of mutations associated with the BCR signaling pathway; this finding deserves further investigations.

There are other noteworthy findings of this study. The higher incidence of DDR pathway mutations in TC patients is consistent with their elevated TMB, suggesting that TC patients are more likely to benefit from immunotherapy. TET-related mutations were also enriched in cytokine-related pathways. As reported previously, cytokines are possibly associated with the development of autoimmune diseases, such as myasthenia gravis, in patients with TETs [45, 46]. Cytokines have a wide range of functions and are also involved in the regulation of hormonal signaling, including parathyroid hormone, thyroid hormone, and insulin secretion pathways [40, 47, 48]. Thus, cytokines are not only closely associated with autoimmune diseases, but they may also be involved in the dysregulation of hormonal pathways in tumors; this issue deserves further attention. We also observed various tumor-related chromosomal amplification or deletion regions in TETs, such as 10p12.31, 11q13.3, and 19p13.3; these regions show a relationship with meningiomas, breast cancer, and melanomas, respectively [49–51]. The presence of multiple CNV regions in TETs is a valuable research direction that could facilitate the diagnosis and treatment of these tumors.

Due to the rarity of TETs, the number of WES studies in this field remains limited. Previous investigations have reported alterations in cancer-related pathways such as PI3K-Akt signaling, which is consistent with our findings. However, most studies have primarily focused on individual genetic alterations without systematically integrating these mutations or correlating them with the clinical manifestations of TETs [52–56]. Our study specifically focuses on alterations in hormone-related pathways and their potential association with PNS in TETs, thereby providing insights that may contribute to improved diagnosis and treatment strategies. The present study also has some limitations. Because of the rarity of TETs, it is difficult to readily obtain cases for investigation; consequently, the present study had a small sample size. This also caused an imbalance in the number of TC and THYM cases in this study. Therefore, we conducted separate preliminary subgroup analyses for TC and THYM. In future studies, we will attempt to increase the sample size to further validate the results. Additionally, because of the lack of established in vitro and in vivo models of TETs, we could not conduct further functional validation. Furthermore, due to the limited sample size, relying entirely on adjusted statistical analysis results would lead to the omission of some tumor- and hormone-related pathways with biological and clinical significance. Therefore, in this study, we primarily present analyses based on *p*-values to intuitively demonstrate potential research clues. The statistical findings have been supplemented with FDR correction analysis, and adjusted *p*-values for all pathways are provided in supplementary tables (Table S1). Pathways showing significance only in terms of *p*-value but remaining nonsignificant following FDR correction require cautious interpretation. In future studies, these findings should be further validated with an expanded sample size to improve the robustness and clinical generalizability of our results. Despite these limitations, we hope that the results of this study could guide further investigations related to TETs.

In summary, we analyzed TETs at the genetic level by using WES technology. We found that genetic alterations in TETs were enriched in multiple hormone-related pathways, including thyroid hormone, parathyroid hormone, insulin secretion, and Cushing’s syndrome. Meanwhile, patients with PNS exhibited more pronounced alterations in these hormone-related pathways. We also identified an interesting gene mutation, *RHPN2*, which may play a potential role in TETs and may be associated with the disruption of hormonal pathways. We analyzed the possible role of hormones in TETs at several levels and explored potential links between hormones and other gene mutations. We hope that the present research generates new ideas to conduct further investigations on the diagnosis and treatment of TETs at the hormonal level. We also expect that this study could guide the development of novel hormonal interventions to assist antitumor therapy, reduce PNS occurrence, and improve the quality of life and survival of patients with TETs.

## Supplementary Information

Supplementary Material 1.

Supplementary Material 2.

Supplementary Material 3.

## Data Availability

Sequence data that support the findings of this study will be deposited in the Genome Sequence Archive (GSA) and are available from the corresponding author upon reasonable request.

## Abbreviations

CNV: copy number variation
DDR: DNA damage repair
FFPE: formalin-fixed paraffin-embedded
GISTIC: Genome Identification of Significant Targets in Cancer
ICIs: immune checkpoint inhibitors
ImmPort: Immunology Database and Analysis Portal
KEGG: Kyoto Encyclopedia of Genes and Genomes
MG: myasthenia gravis
MsigDB: Molecular Signatures Database
MSI: microsatellite instability
PD-1: programmed death 1
PD-L1: programmed death ligand-1
PFS: progression-free survival
PNS: paraneoplastic syndromes
TC: thymic carcinomas
TETs: thymic epithelial tumors
THYM: thymomas
TMB: tumor mutation burden
VAF: variant allele frequency
WES: whole-exome sequencing
