# Supplementary material for: Genetic alterations and aberrant hormonal pathways in thymic epithelial tumors
Source: BMC Cancer. 2025 Dec 12;26:98. doi: 10.1186/s12885-025-15455-4 (PMC12821246; doi:10.1186/s12885-025-15455-4)
Supplement: Supplementary file 3 — Supplementary Material 3. [file 12885_2025_15455_MOESM3_ESM.zip › Supplementary figure legends.docx]

**Figure S1.** Differences in gene enrichment between *RHPN2* mutant and wild type. (A) Analysis based on the gene set derived from the Kyoto Encyclopedia of Genes and Genomes (KEGG) database, which shows the pathways with p.adjust < 0.2 following FDR correction. (B) Analysis based on the gene set derived from the Hallmark database.

**Figure S2.** Gene enrichment of thymic epithelial tumors (TETs) in 24 patients. (A) Analysis based on the gene set derived from the Kyoto Encyclopedia of Genes and Genomes (KEGG) database, which shows the pathways with p.adjust < 0.2 following FDR correction. (B) Analysis based on the gene set derived from the Hallmark database.

**Figure S3.** Differences in gene enrichment between thymic carcinoma (TC) and thymoma (THYM). (A) Analysis based on the gene set derived from the Kyoto Encyclopedia of Genes and Genomes (KEGG) database, which shows the pathways with p.adjust < 0.2 following FDR correction. (B) Analysis based on the gene set derived from the Hallmark database.
