# Supplementary figures and images for: Genetic alterations and aberrant hormonal pathways in thymic epithelial tumors
Source: BMC Cancer. 2025 Dec 12;26:98. doi: 10.1186/s12885-025-15455-4 (PMC12821246; doi:10.1186/s12885-025-15455-4)

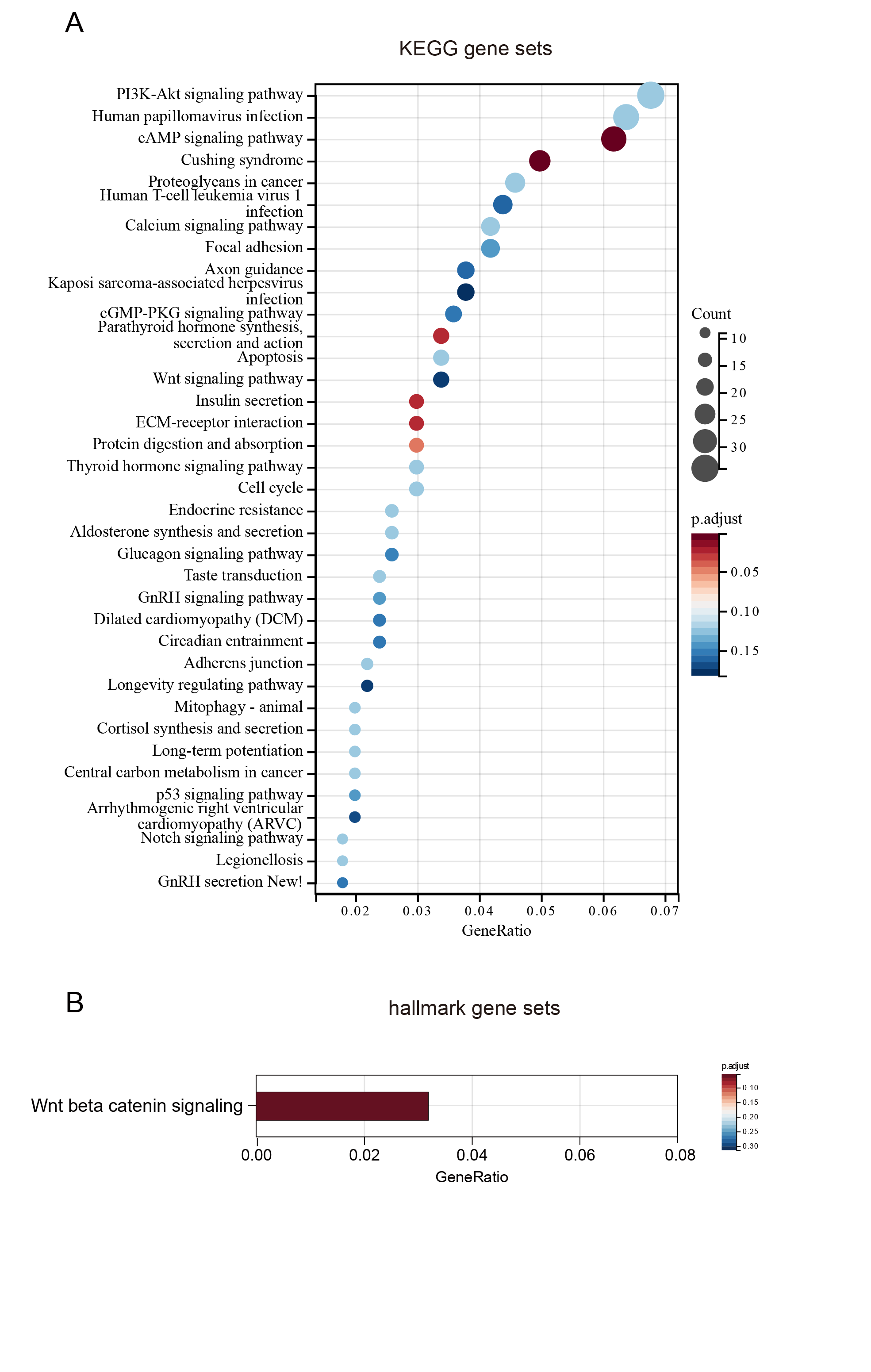

Supplement: Supplementary file 3 — Supplementary Material 3. [file 12885_2025_15455_MOESM3_ESM.zip › figureS2.tif]

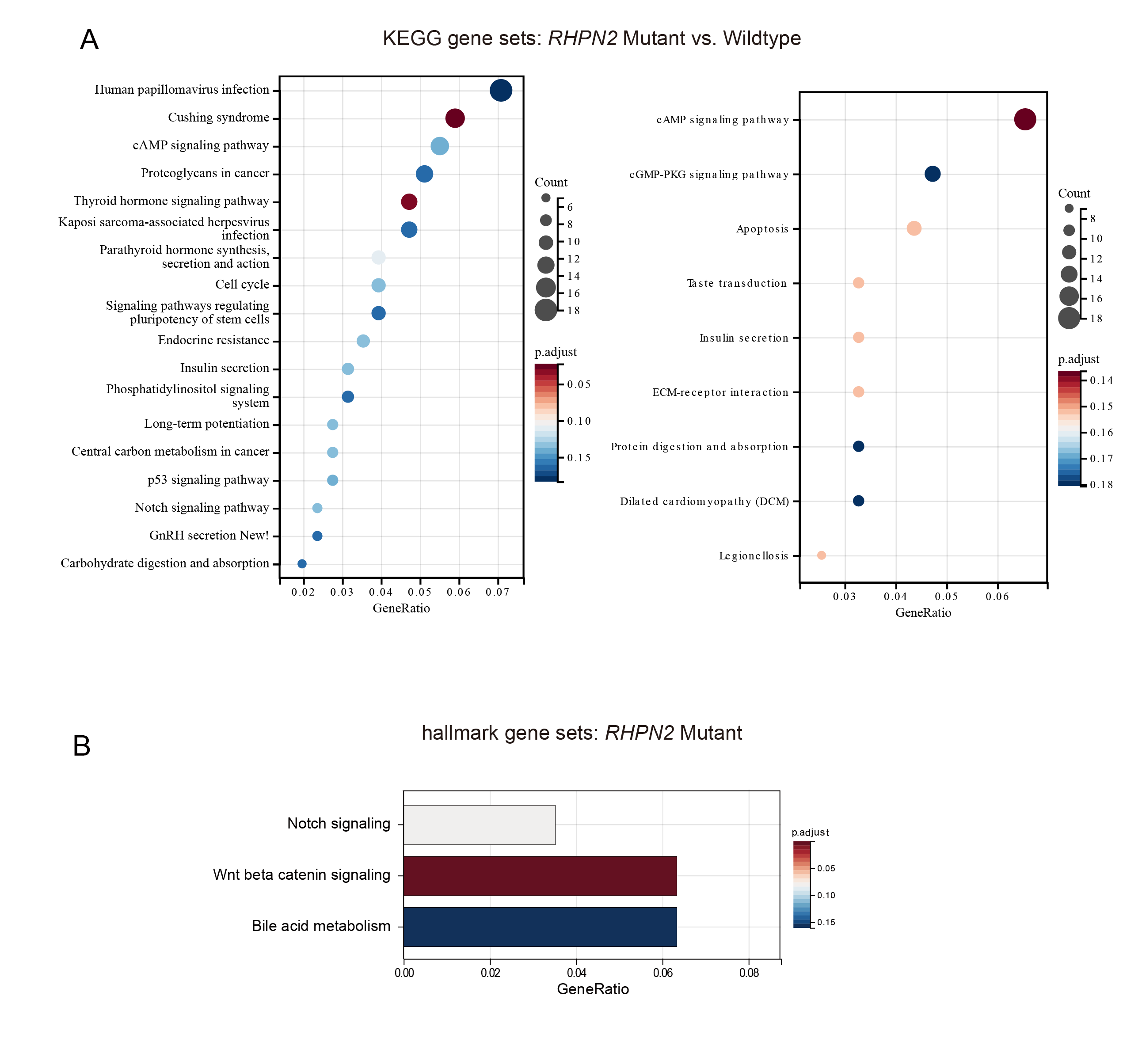

Supplement: Supplementary file 3 — Supplementary Material 3. [file 12885_2025_15455_MOESM3_ESM.zip › figureS1.tif]

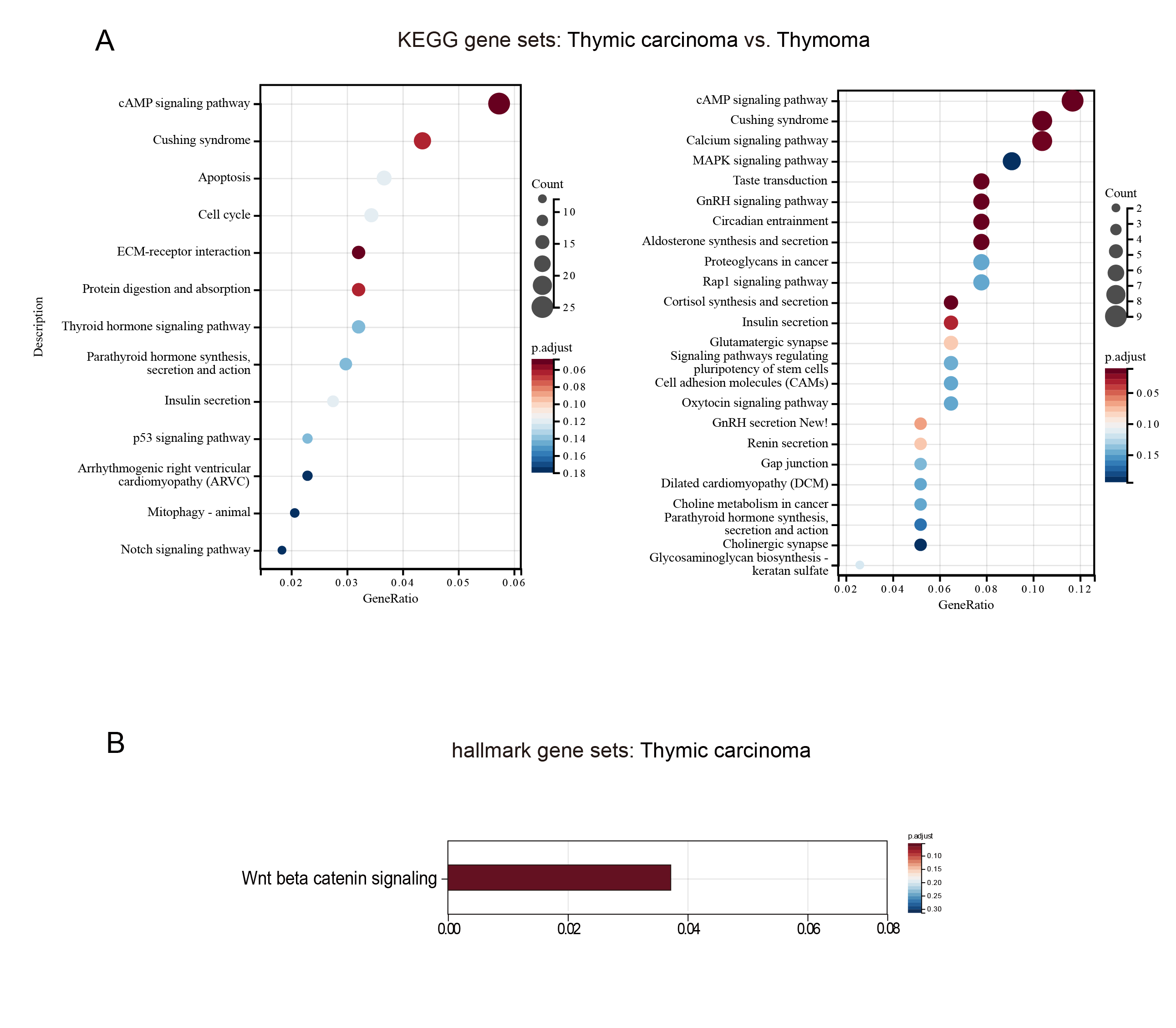

Supplement: Supplementary file 3 — Supplementary Material 3. [file 12885_2025_15455_MOESM3_ESM.zip › figureS3.tif]
